# The British Congress on Tuberculosis

**Published:** 1901-09

**Authors:** 


### EDITORIALS.

### THE BRITISH CONGRESS ON TUBERCULOSIS.

The celebrity of Prof. Koch's name and the craving for sensationalism on the part of many of the readers of the public press, which the latter subserves to, overshadowed and hid from view the vast amount of valuable work which was presented to the congress.

That of our own colleagues was, in its scientific and practical exposition, so important that the veterinary profession has cause to look with pride to the men who represented it.

The report of this congress will be a standard authority upon the subject of tuberculosis. The various papers form a complete index upon all matters connected with the disease.

Among the more important papers:

"Tuberculosis and the Milk Supply," by John A. W. Dollar, M.R.C.V.S., gives a review of the experiments of Villemin, Chauveau, Klebs, Bollinger, Crookshank, Ollivier, Johne, Hermsdorf, Leonhardt, Sonntag, Stang, Schuppenhauer, Demme, and others. It covers the reports on the chemical and microscopical examination of milk by Obermüller, Rabinowitsch, Jäger, Petri, Bang, of Copenhagen; Fiorentini, of Milan; Peters, of Boston, Hamilton, Boyce, Moorhead, and Delapine.

The geographical distribution and percentages are given from the reports of Mr. C. Hunting, in England, and from official reports in Europe, America, and Australia.

The post-mortem work of Boltz, Grawitz, Biedert, Behrens, Wyss, and Von Eisenhardt is given.

The paper ends with a résumé on prevention and diagnosis.

"The Diagnosis of Tuberculosis in Animals During Life," by Prof. Dewar, F.R.C.V.S., Principal of the Royal Veterinary College, Edinburgh, is most practical in reviewing the to-day frequently neglected symptoms to be obtained by physical diagnosis and in criticising the errors made in using the tuberculin test.

"Tubercle Bacilli in Cow's Milk as a Possible Source of Tuberculous Disease in Man," by Prof. John McFadyean, M.B., M.R.C.V.S., is a practical and valuable paper. Prof. McFaydean makes an interesting summary of Koch's work, the last of which, his proposition given a few days before, at the opening of the congress, had caused serious consideration.

Dr. Brouardel, of Belguim, gave a synopsis of the legislation for the prevention of the spread of tuberculosis.

"Legislation Suggested for Controlling and Eradicating Tuberculosis in Animals," by Duncan McEachran, of Magill University, Montreal, is a most practical paper, dealing with existing laws and proposing reasonable additions in legislation.

The discussions were entered into by many of the most celebrated veterinary investigators, and are well worth reading and study.

